# Biogenic selenium and its hepatoprotective activity

**DOI:** 10.1038/s41598-017-13636-1

**Published:** 2017-11-15

**Authors:** Baozhen Li, Dan Li, Weixin Jing, Jinhua Fan, Hans-Uwe Dahms, Shao-Chin Lee, Lan Wang

**Affiliations:** 1 School of Life Science, Shanxi University, Taiyuan, 030006 People’s Republic of China; 2 Department of Biomedical Science and Environmental Biology, Kaohsiung Medical University, Kaohsiung, 80708 Taiwan; 3 Research Center for Environmental Medicine, Kaohsiung Medical University, Kaohsiung, 80708 Taiwan

## Abstract

Elemental selenium nanoparticles (SeNPs) have multiple biological activities. In this study, we investigated the protective effects of biogenic SeNPs (BioSeNPs) on CCl_4_-induced liver damage in mice. The results showed that: (i) when compared to sodium selenite (SS), BioSeNPs has a similar tissue distribution after intragastrical administration to mice; (ii) BioSeNPs and SS showed comparable efficacy in increasing the activities of glutathione peroxidase and thioredoxin reductase in liver cell lines, mice blood and liver; (iii) pretreatment with BioSeNPs inhibiting the elevation of activities of various enzymes significantly which included aspartate aminotransferase, alanine aminotransferase, alkaline phosphatase, lactate dehydrogenase and liver lipid peroxide (p < 0.05 or p < 0.01) in CCl_4_-treated mice; (iv) activities of antioxidant enzymes (superoxide dismutase, catalase, and glutathione peroxidase) were significantly increased (p < 0.05 or p < 0.01) after a pretreatment with BioSeNPs in CCl_4_-treated mice; (v) histopathological damages in the liver from CCl_4_-treated mice were ameliorated by a pretreatment with BioSeNPs. In conclusion, these results have shown that BioSeNPs is able to protect the liver from CCl_4_-induced hepatic damage via increasing the antioxidant capacity and inhibiting oxidative damage. BioSeNPs may have the potential to be used as a trace element food supplement inducing antioxidant bioactivities.

## Introduction

Selenium (Se), one of the essential trace elements, has multiple beneficial effects for human health, such as acting as antioxidant, prevention of cancer initiation, growths, and metastasis without toxic side effect^1–4^. However, the element and its derivates have different bioavailability and biological activities^5,6^. Among the various forms of Se, Se nanoparticles (SeNPs) are envisaged widely in biomedicine due to their high bioavailability and diverse biological activities^7,8^.

SeNPs can be synthesized through physical, chemical, and biological methods^9,10^. Se^0^ has been considered as biologically inactive until Zhang^11^ showed that, compared to sodium selenite (SS), chemically synthesized SeNPs (ch-SeNPs) were less toxic to cells and tissues, while maintaining similar antioxidant and bioavailability properties. In fact, in animal models, ch-SeNPs were proven to be effective antioxidants without notable cytotoxicity which is a typical side effect of other chemical forms of Se^12^. Therefore, SeNPs have attracted much attention to be used as potential nutrition supplementation^7,9,13^.

Nevertheless, the preparations of SeNPs using physical or chemical methods require high temperature, extreme pH, and harmful chemicals, which are proven to be very expensive and can cause environmental pullution^14,15^. Biogenic methods provide a renewable, clean, nontoxic, and environmentally friendly procedure for the synthesis of SeNPs^10,16,17^. Biological synthesized SeNPs (BioSeNPs) are known from bacteria, fungi, yeasts, and plants^9,10,15,18^. Among them, bacteria are the first choice for the biosynthesis of particles, because of their rapid extracellular production, ease and inexpensive culture, and ease of post-processing^19^. In recent years, several different bacteria have been reported to be capable of synthesizing SeNPs, such as *Rhizobium selenitireducens* strain B1^20^, *Escherichia coli* ^21^, *Pseudomonas fluorescens* K27^22^, and *Bacillus cereus* ^23^. However, elemental Se particles produced by some bacterial strains have a very low bioavailability over extended periods of time^24,25^. BioSeNPs demonstrated antioxidant and anticancer activities in recent studies without mentioning its bioavailability in mice^5,26,27^.

Photosynthetic bacteria (PBS) are rich in protein, carotenoids, biological cofactors, and vitamins^28^. Studies have shown that PBS is suitable as a healthy food supplement for humans and animals^15^. Recently, our group reported that strain N of *Rhodopseudomonas palustris* (*R*. *palustris*), a typical purple non-sulfur bacterium, can reduce Se^4+^ to red elemental Se at room temperature under conventional culture conditions^29^. Thus, this bacterial strain provides a candidate supplement for the biosynthesis of SeNPs.

Carbon tetrachloride (CCl_4_) is a chemical agent that induces liver damage. CCl_4_ causes oxidative stress and lipid peroxidation by cytochrome P4502E1-mediated generation of high activity radicals, eventually leading to liver cell necrosis^30^. In recent years, the antioxidant and hepatoprotective activity of SeNPs have been reported. However, these reports focused on the study of SeNPs obtained from physicochemical synthesis^12,31^. The protective effect of BioSeNPs on liver damage has not been reported. Therefore, the purpose of our study is to verify that BioSeNPs is able to protect the liver from CCl_4_-induced hepatic damage via increasing the antioxidant capacity and inhibiting oxidative damage.

Here, we investigated the protective effect of BioSeNPs from strain N on CCl_4_-induced liver damage and the underlying mechanisms of this protection.

## Results

### Transformation of Se^4+^ to SeNPs

**Figure 1:**
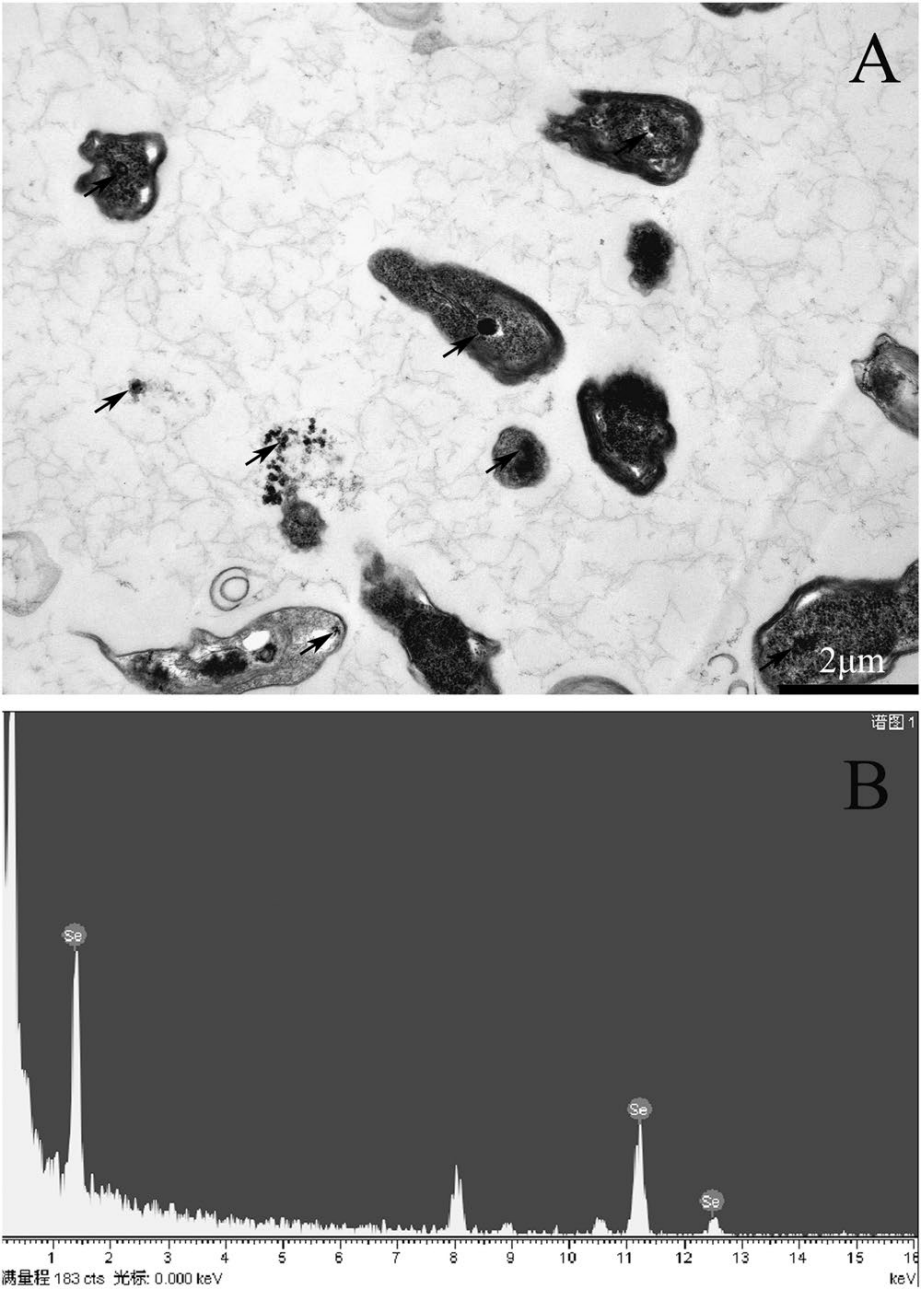
Characterization of SR: (**A**) TEM micrographs, and (**B**) EDX spectrum of the particles.

An electron micrograph of cells and Se-containing particles obtained from the culture medium was amended with 3.0 m mol/L SS is shown in Fig. 1A. Spherical SeNPs of various sizes had formed on the inside and the surface of *R*. *palustris* cells. The particle sizes ranged from 80 to 200 nm as shown in our previous publication^29^. Furthermore, Se-containing particles were analyzed by using energy dispersive X-ray spectrum (EDX) analysis, the electron-dense particles produced specific Se absorption peaks at 1.37 keV (peak SeLa), 11.22 keV (peak SeKa), and 12.49 keV (peak SeKb) (Fig. 1B). These results indicate that Se^4+^ was reduced to red elemental SeNPs by *R. palustris*.

### Induction of Se-containing enzymes in HepG2 cells

**Figure 2:**
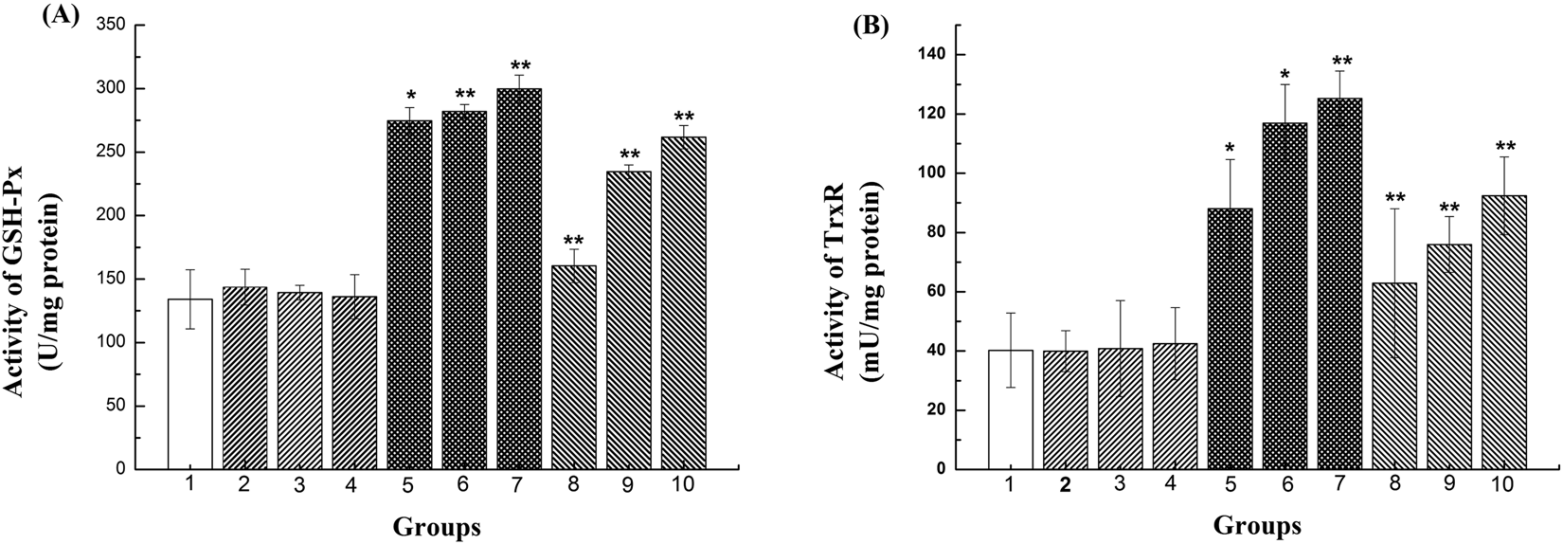
Se-containing enzyme activities induced by SR and SS in HepG2 cells. Data show means ± SD (n = 3). ***Significantly difference compared to group1 (*p < 0.05, **p < 0.01).

In this study, we added 10, 20, and 50 nM SS and SeNPs-enriched *R. palustris* (SR) to compare their effect on inducing Se-containing enzymes in HepG2 cells. As shown in Fig. 2, the GSH-Px, and TrxR activities were significantly increased by SR or SS after 4 days of Se supplementation (p < 0.05) in a dose dependent manner. Pretreatment with the bacteria alone did not modify the activities of the enzymes.

### Se accumulation in the tissue

**Figure 3:**
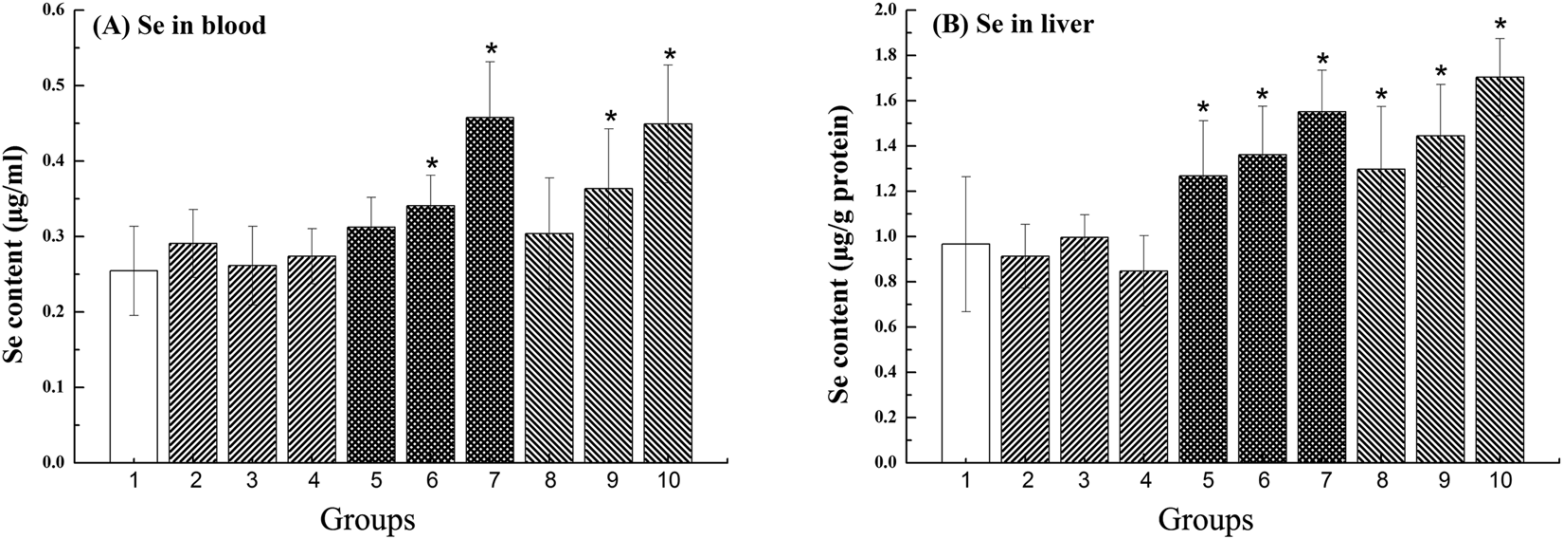
Se accumulation in mice supplemented with SR and SS: (**A**) Se in blood; (**B**) Se in liver. *Significantly difference compared to group1 (*p < 0.05).

Se accumulation in blood and liver occurs in enzymatic and non-enzymatic proteins as well as in inorganic ionic forms. After 7 days of Se supplementation, compared to those in the group1, the blood (Fig. 3A) and liver (Fig. 3B) samples from mice pretreated with SR or SS had significantly higher levels of Se (p < 0.05). In contrast, bacteria alone did not change the Se concentration of the tissues (Fig. 3A,B). There was no significant difference in Se accumulation between groups 5–7 and groups 8–10.

### Induction of Se-containing enzymes in mice

**Figure 4:**
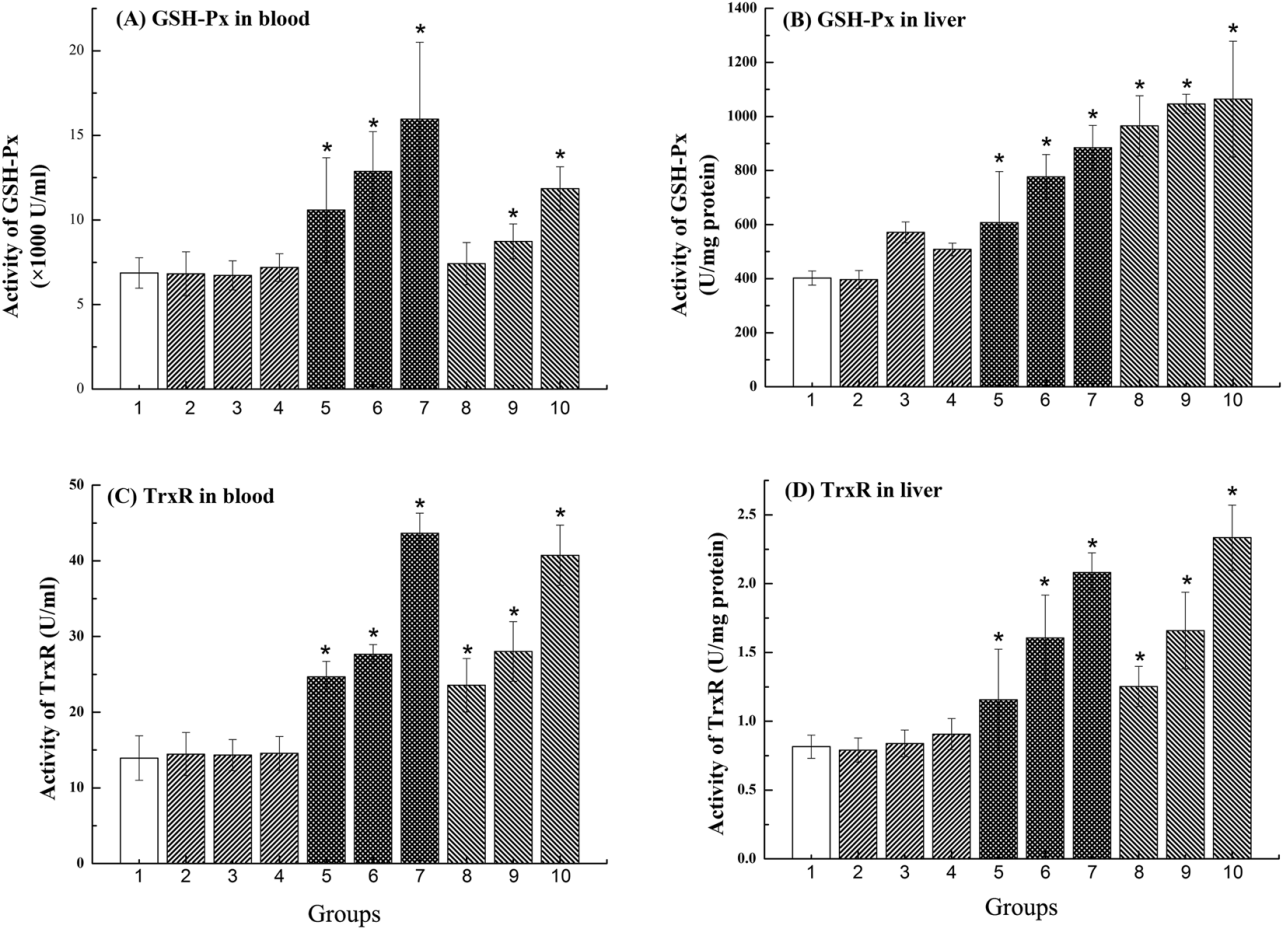
Se-containing enzymes in mice supplemented with SR and SS: (**A**) GSH-Px in blood; (**B**) GSH-Px in liver; (**C**) TrxR in blood; (**D**) TrxR in liver. *Significantly difference compared to group1 (*p < 0.05).

As shown in Fig. 4, glutathione peroxidase (GSH-Px) activities of blood (Fig. 4A) and liver (Fig. 4B) were significantly increased by SR or SS. Similar to the GSH-Px activity, the activities of thioredoxin reductase (TrxR) in mice blood (Fig. 4C) and liver (Fig. 4D) also showed a significant increase in the groups 5–7 and groups 8–10 after 7 days of Se supplementation (p < 0.05). Pretreatment with the bacteria alone did not modify the activities of the enzymes.

### Effects of SR on hepatic function in CCl4-treated mice

Several hepatic enzymes in serum such as alanine aminotransferase (ALT), aspartate aminotransferase (AST), alkaline phosphatase (ALP), and lactate dehydrogenase (LDH) were used as biochemical markers for early acute hepatic damage. The levels of ALT, AST, ALP, and LDH in the serum were measured. In comparison with group1, the CCl_4_-treated mice in group3 had higher levels of ALT (21062.51 ± 1217.47 U/L vs. 15.6 ± 2.14 U/L, p < 0.001), AST (3198.51 ± 313.323 U/L vs. 59.54 ± 5.51 U/L, p < 0.001), ALP (16.4 ± 0.26 U/L vs. 7.56 ± 0.32 U/L, p < 0.01), and LDH (649096.39 ± 9958 U/L vs. 19122.2 ± 1395.17 U/L, p < 0.001) (see Table 1). High dose of SR alone did not cause an elevation of the activities of serum ALT, AST, ALP, and LDH. As expected, this CCl_4_-induced increase was effectively attenuated by pretreatment with SR (p < 0.05 or p < 0.01) in a dose-dependent manner. Pretreatment with bacteria alone was unable to inhibit the increase in the enzymatic activities caused by CCl_4_ (Table 1).

**Table 1:** Effect of SR on serum enzyme activities in mice: ALT, AST, ALP, LDH.

Test items	Group1	Group2	Group3	Group4	Group5	Group6	Group7	Group8	Group9
ALT (U/L)	15.60 ± 2.14	15.08 ± 1.26	21062.5 ± 1217.5^**###**^	23782 ± 2795.4	19676 ± 2619.9	19848 ± 2544.8	13701 ± 1027.9*	13576 ± 1296.0**	12284 ± 1177.8**
AST (U/L)	59.54 ± 5.51	60.07 ± 3.74	3198.5 ± 313.3^**###**^	3112.6 ± 256.5	2666.7 ± 194	2504.6 ± 117.5	2090.1 ± 91.60*	1545.2 ± 248.5**	1466.2 ± 198.3**
ALP (U/L)	7.56 ± 0.32	9.97 ± 0.97	16.40 ± 0.26^**##**^	15.35 ± 0.34	14.14 ± 0.37	14.90 ± 0.79	11.43 ± 0.50*	10.78 ± 1.14*	8.00 ± 0.72**
LDH (U/L)	19122.2 ± 1395.2	17538.7 ± 737.2	649096.4 ± 9958^**###**^	662650 ± 18500.9	613730 ± 28290	624100 ± 23274.9	559720 ± 6494.2*	558730 ± 11230.3*	529780 ± 34897.9**

^**##**^ *P* < 0.01, ^**###**^ *P* < 0.001, compared to group1; *P < 0.05,** *P* < 0.01, compared to group3.

### Effects of SR on antioxidant enzyme activities in liver from CCl_4_-treated mice

**Figure 5:**
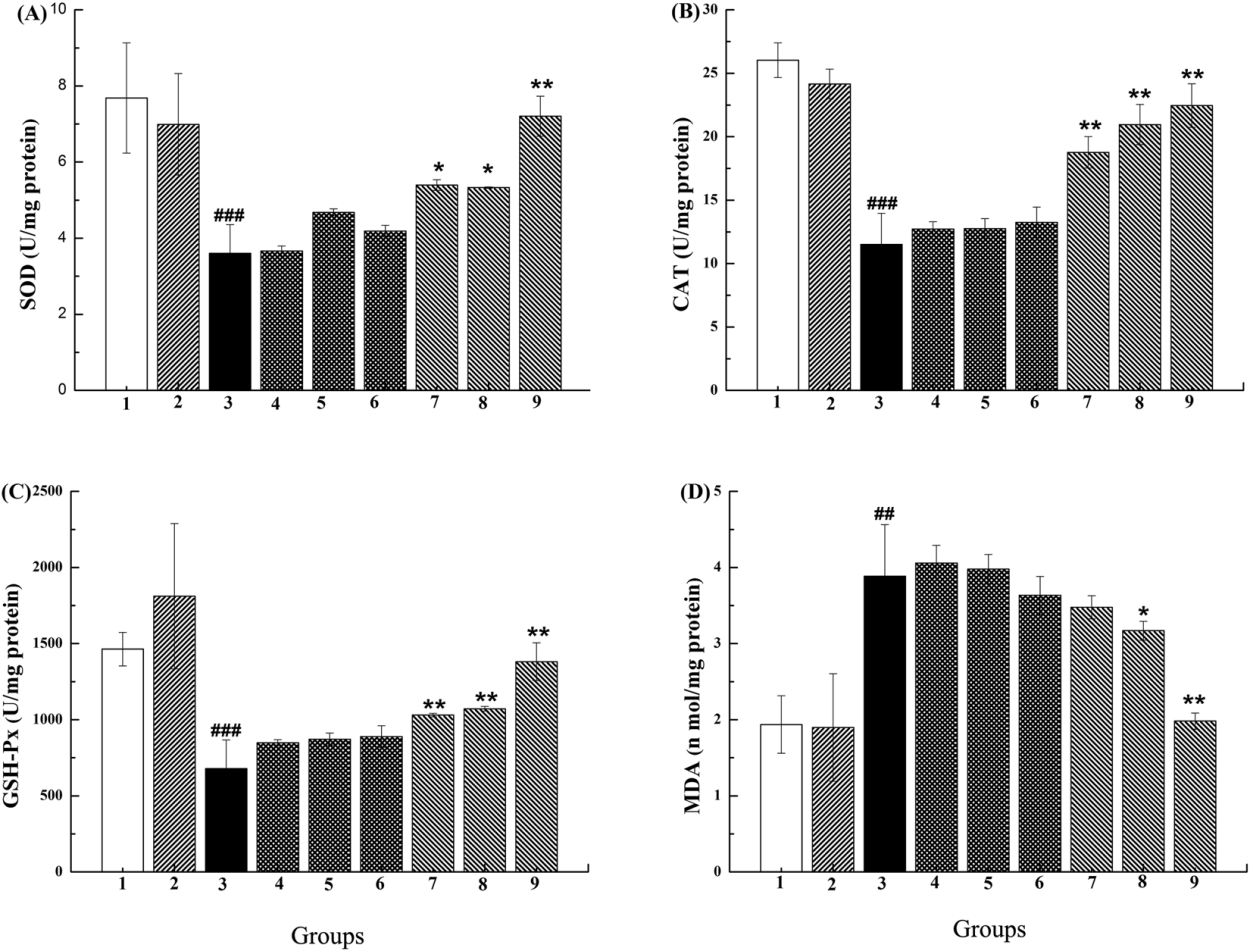
Effects of SR on the activities of liver SOD (**A**), CAT (**B**), GSH-Px (**C**), and the level of liver MDA (**D**) in CCl_4_-treated mice. ^##, ###^Significant differences compared to group1 (^##^p < 0.01, ^###^p < 0.001). *^,^**Significant differences compared to group3 (*p < 0.05, **p < 0.01).

The hepatic antioxidant enzyme activities [superoxide dismutase (SOD), catalase (CAT), and GSH-Px] were measured to investigate the effect of SR on the antioxidant capacity. The CCl_4_-treated mice in group 3, the activities of liver SOD, CAT, and GSH-Px were significantly decreased (p < 0.001) (Fig. 5A–C). However, high dose of SR alone did not cause a decrease of the activities of liver SOD, CAT, and GSH-Px. Pretreatment with SR, but not the bacteria alone, was able to significantly prevent the inhibition effects of CCl_4_ on the enzymes (p < 0.05 or p < 0.01), in a dose-dependent manner (Fig. 5A–C).

### Effects of SR on lipid peroxidation in liver tissue from CCl4-treated mice

Hepatic levels of malondialdehyde (MDA) were assessed as an indicator of lipid peroxidation in the tissue. In the group 3, MDA levels in liver were significantly increased (p < 0.001) in mice treated with CCl_4_ (Fig. 5D). However, high dose of SR alone did not cause an elevation of liver levels of MDA. Pretreatment with SR, at the medium-dose group and high-dose group, significantly prevented an increase in the levels of MDA (p < 0.05 or p < 0.01) in the mice treated with CCl_4_. In contrast, pretreatment with *R. palustris* alone did not prevent an increase in the MDA levels in the mice treated with CCl_4_.

### Effects of SR on histopathological changes in CCl4-induced hepatotoxicity

**Figure 6:**
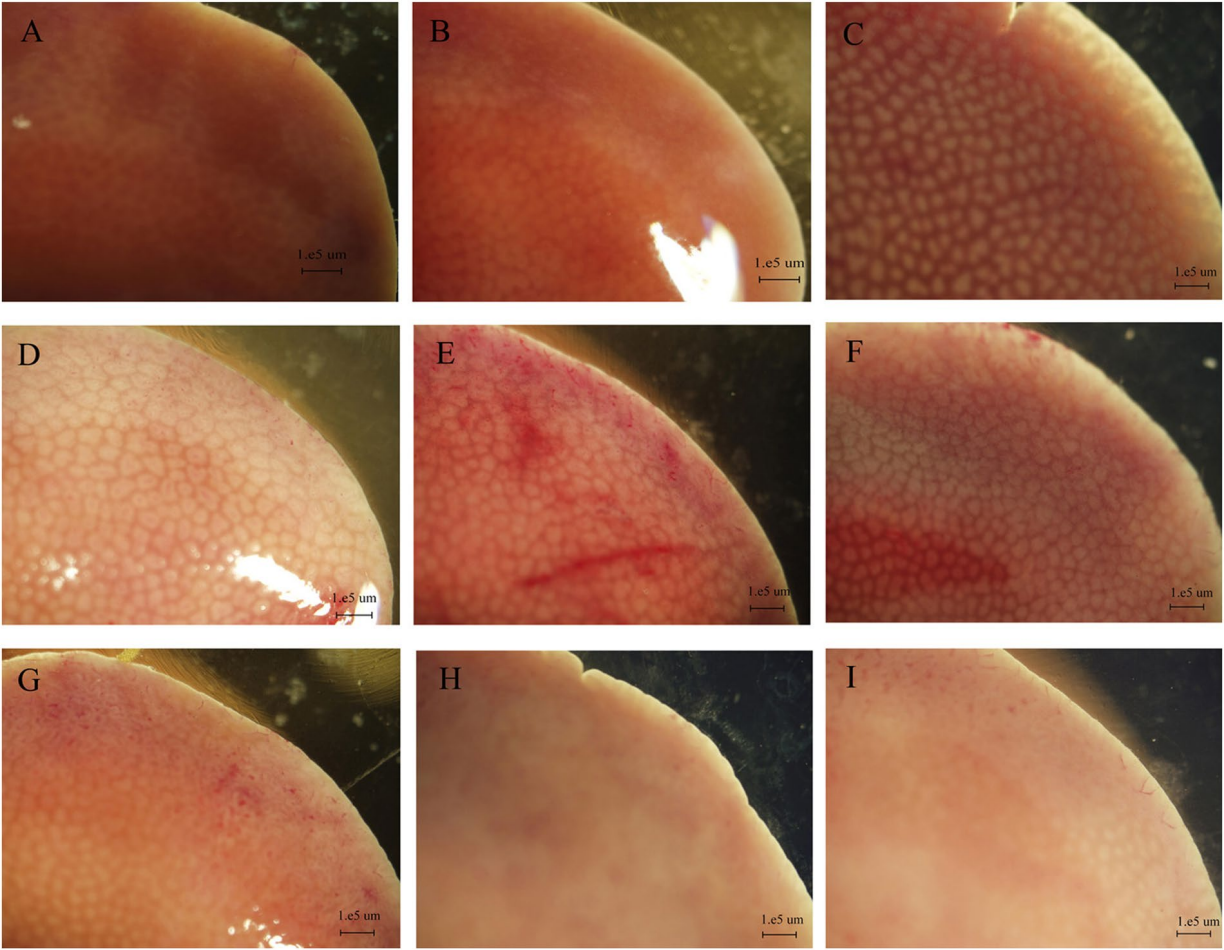
Effects of SR on CCl_4_-induced liver damage. Liver tissues were observed under light microscopy. (**A**) group1; (**B**) group2; (**C**) group3; (**D**) group4; (**E**) group5; (**F**) group6; (**G**) group7; (**H**) group8; (**I**) group9.

In this study, the surface of liver in the control group was dark red, luster, and smooth, with tidy edges as revealed by stereo light microscopy (Fig. 6A). Mice pretreated with a high dose of SR alone did not cause abnormalities in the liver (Fig. 6B). When treated with CCl_4_ alone, the liver surface was grey, lacked lusters with irregular margins, and had bleeding spots in group3 (Fig. 6C). Pretreatment with SR attenuated the bleeding symptom substantially at all the tested concentrations (Fig. 6G–I), especially when the dosage increased to 200 µg Se/kg BW. Moreover, pretreatment with *R. palustris* did not attenuate the bleeding symptom substantially at all the tested concentrations (Fig. 6D–F).

**Figure 7:**
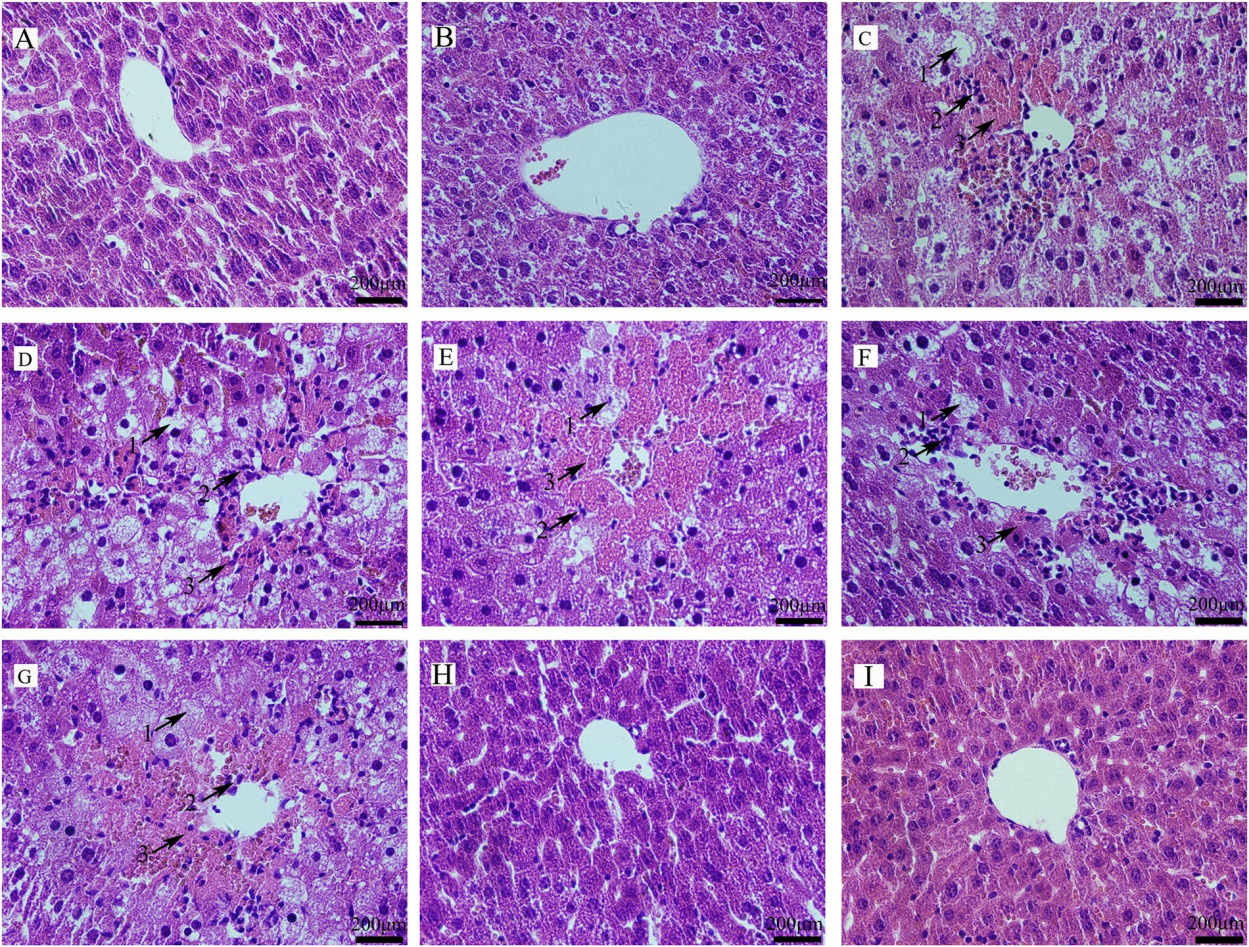
Effects of SR on CCl_4_-induced liver damage. Sections were stained with haematoxylin-eosin and observed under light microscopy. (**A**) group1; (**B**) group2; (**C**) group3; (**D**) group4; (**E**) group5; (**F**) group6; (**G**) group7; (**H**) group8; (**I**) group9. 1: ballooning degeneration; 2: inflammatory cell infiltration; 3: cell necrosis.

When mice were treated with CCl_4_ in group 3, CCl_4_ caused extensive changes in liver morphology, including severe cellular degeneration, hepatocyte necrosis, and cytoplasm vacuolization (Fig. 7C). The hepatic cellular architecture of mice pretreated with a high dose of SR alone (Fig. 7B) was similar to those in the group 1 (Fig. 7A) with physiological saline. However, the histopathological damages in the liver from CCl_4_-treated mice were ameliorated by the pretreatment with SR in a dose-dependent manner (Fig. 7G–I); SR at 200 µg Se/kg BW was able to entirely block the tissue damages by CCl_4_, resulting in well-preserved cytoplasm, prominent nuclei and legible nucleoli (Fig. 7I). Pretreatment with the *R. palustris* did not show an effective protection against CCl_4_-induced liver damage (Fig. 7D–F).

## Discussion

Absorption and utilization of Selenium in an organism is a prerequisite for its biological function. In 2004, Zhang *et al*. found that ch-SeNPs from 5 to 200 nm had similar efficacy compared to SS and had no size effect in the induction of seleno-enzymes in both cultured cells and mice. But, ch-SeNPs above 200 nm showed less absorption and utilization^12,32^. And, some reports have indicated that SeNPs about 300 nm obtained from bacteria has very low absorption and utilization compared to SS^24,25,33^. In our study, SeNPs from *R. palustris* range from 80–200 nm (Fig. 1A) had similar efficacy in Se accumulation and effects on GSH-Px as well as TrxR activity in both cultured cells and mice. Similar to ch-SeNPs, BioSeNPs may also have size effect in absorption and utilization.

Some studies have shown that different forms of selenium can increase GPx and TrxR activity in blood and liver^11,12,32^. Our results demonstrated that the SR improves the blood GSH-Px compared to SS, and GSH-Px levels were increased significantly by SS compared to SR in the liver. However, this trend was not observed for TrxR (Fig. 3). This may be due to the fact that GSH-Px activity changes with concentration effect and tissue differences^34^ and the activity of TrxR is no very much tissue specific^35,36^.

CCl_4_-induced liver injury is the best system of xenobiotic-induced hepatotoxicity and a commonly used model for the screening of liver protection drugs^37,38^. Liver damage induced by CCl_4_ is associated with severe lipid peroxidation, depletion of the antioxidant capacity, and damages to cell membranes as well as organelles^39–41^. In our study we showed for the first time that treatment with BioSeNPs from *R. palustris* can significantly prevent CCl_4_-induced acute liver damage (Fig. 4).

Serum enzymes, such as ALT, AST, ALP, and LDH are generally recognized as bio-chemical markers of liver injury, and they will exhibit increased activity when hepatocytes are necrotic^42–44^. The present study showed that the activities of ALT, AST, ALP, and LDH in group 3 were maximally increased 1350-, 43.7-, 2.2- and 33.9-fold compared to the activities in the group 1. The results suggested serious hepatocellular damage (Table 1). Supplementation of BioSeNPs significantly attenuated an increase in the enzymatic activities in the serum. Compared with group 3, the activities of ALT, AST, ALP, and LDH in the group 9 were decreased by 41.68%, 54.16%, 51.24% and 18.38%. Histopathological examination of the liver sections in CCl_4_-treated mice revealed extensive liver injuries, characterized by severe cellular degeneration, hepatocyte necrosis, and cytoplasmic vacuolization (Figs 6C and 7C). However, liver histopathological lesions were significantly ameliorated by the pretreatment with BioSeNPs, which was consistent with the results of the biochemical analysis (Figs 6G–I and 7G–I).

Hepatoprotective effects may be associated with an antioxidant capacity to scavenge reactive oxygen species (ROS)^45^. The intracellular redox balance depends on ROS production and antioxidant-defense system that includes enzymatic antioxidants such as SOD, CAT, and GSH-Px in cells^30,46^. Therefore, enzymatic antioxidant activities and the inhibition of free-radical generation are important in terms of protecting the liver from CCl_4_-induced damage^30^. To show the effect of BioSeNPs on oxidative stress-mediated hepatic injury, we have determined several oxidative stress indicators, including SOD, CAT, GSH-Px, and MDA. Consistent with reported results^47^, we found that supplementation of SeNPs from *R. palustris* could significantly increase the activities of SOD, CAT and GSH-Px and markedly decreased the MDA levels in the liver of CCl_4_-treated mice. The activities of SOD, CAT and GSH-Px in group 9 maximally increased 2.00-, 1.95-, and 2.03-fold. MDA levels decrease by 49.00% compared to group 3 (Fig. 5). This suggests that our BioSeNPs increased the antioxidant capacity of liver to counteract oxidative stress caused by CCl_4_.

In conclusion, our SeNPs from strain N of *R. palustris* had a similar efficacy in Se accumulation and Se-containing enzymes activities in both cultured cells and mice to SS. It showed no notable toxicity *in vivo* mice. Moreover, it can protect mice from CCl_4_-induced hepatic damage by increasing the antioxidant capacity to inhibit oxidative stress. The BioSeNPs can be developed as a food supplement for improving human body antioxidant capacity and preventing oxidative damage.

## Methods

### Preparation of SeNPs-enriched and control of *R. palustris*

*R*. *palustris* was cultured according to the method described by Li *et al*.^29^, except that the medium was supplemented with 3.0 m mol/L SS and the strain was incubated at 30 °C in the presence of incandescent light (1500 Lux) at gentle stirring (80 r/min) with magnetic stirrer for 8 days for SeNPs enrichment. Control samples were cultured without SS. During the culture, the Se^4+^ in SS was reduced to form red elemental Se. The bacteria were then collected by centrifugation at 5000 × g for 30 min at 4 °C, washed three times with sterile 0.9% saline solution, and stored at −4 °C until use for the animal study. Bacterial cells were resuspended in 2.5% glutaraldehyde and fixed for 2 h, washed with phosphate buffer (0.2 m mol/L, pH 7.0), and embedded in low-melting-point agarose. Agar blocks (approximately 1 by 1 by 1 mm) were fixed in 1% OsO_4_ in running water for 60 min, dehydrated with ethanol and acetone, and embedded in Epon-Araldite. Sections cut from the Epon-Araldite preparation were contrasted with uranyl acetate and lead citrate as described by Li^29^. The samples were examined using a JEM-1011 transmission electron microscope (JEOL, Tokyo, Japan) with an accelerating voltage of 80 kV. To determine the elemental composition of the nanoparticles, whole bacterial cells were applied to transmission electron microscopy grids, dried at room temperature, and coated with 5 nm of carbon before measurements were obtained. The EDX analysis was performed with a JEM-2010 transmission electron microscope (JEOL, Tokyo, Japan) equipped with a Link-Inca microanalysis system. The dwelling time for the EDX analysis was 100 s.

### Cell Culture and Preparation of Cell Extracts

Human liver cancer HepG2 cells were purchased from Cell Bank of Type Culture Collection of Chinese Academy of Sciences (Shanghai, China). HepG2 were maintained in DMEM medium supplemented with 10% fetal bovine serum, 1% L-glutamine, and 1% penicillin-streptomycin in a humidified incubator with 5% CO_2_ at 37 °C. For enzyme assays, cells were seeded in 10-cm dishes, and normally reached about 50% confluence at the time of Se-supplementation. Cells were treated with SR and SS from a prepared stock solution in PBS, added to the culture medium to obtain final concentrations indicated as in each experiment (in accordance with Table 2). Equivalent volumes of PBS were used as a vehicle. The doses of Se were chosen according to the report by zhang^32^. Cells were harvested after 4 days incubation with Se. Each treatment contained three replications. Adherent cells were washed twice with phosphate-buffered saline (PBS) and harvested using trypsin/EDTA. Cell extracts for measurement of enzyme activities were obtained by sonication in 0.1 M Tris-HCl at pH 7.4, containing 0.1% digitonin and then centrifuged at 15,000 *g* at 4 °C for 15 min to obtain supernatants. The activities of GSH-Px (Nanjing Jian Cheng, Nanjing, China) and TrxR (Beijing Solarbio, Beijing, China) in cell were estimated using their kits by spectrophotometry, according to the manufacturer’s instructions. Protein levels were determined by the Bradford assay with bovine serum albumin as a standard^48^.

**Table 2:** Activities of GSH-PX and TrxR in HepG2 cells (*n = *3).

Groups	Contents
1	Normal
2	6.0 ng/ml *R. palustris*
3	12.0 ng/ml *R. palustris*
4	30.0 ng/ml *R. palustris*
5	6.0 ng/ml SR (included 10 n mol/L Se)
6	12.0 ng/ml SR (included 20 n mol/L Se)
7	30.0 ng/ml SR (included 50 n mol/L Se)
8	1.73 ng/ml SS (included 10 n mol/L Se)
9	3.46 ng/ml SS (included 20 n mol/L Se)
10	8.65 ng/ml SS (included 50 n mol/L Se)

### Animal model

All animal experiments were approved by the Institutional Committee from China Institute for Radiation Protection (Taiyuan, China) and comply with the Institute for Lab Animals’ guidelines for the humane care and use of laboratory animals. Se-deficient and normal male Kunming mice (body weight: 18–22 g) and low Se diet (<0.02 μg Se/g) were purchased from Shanxi Medical University Animal Centre (Taiyuan, China). The mice were kept in plastic cages (5/each), allowed free access to water and food, and maintained at a 12:12 h light and dark cycle during the study period. The temperature and humidity were regulated at 22 ± 1 °C and 50 ± 10%, respectively. The mice were acclimatized for 1 week before experimental to use.

### Se accumulation and Se-containing enzymes in mice

The SR was re-suspended in 0.9% saline solution to obtain the desired bacterial cell concentrations of 8.438 × 10^8^ CFU/ml. The content of total Se in the SR was 200.0 mg/L, with >99.60% being elemental SeNPs and 0.24% being organic Se, which were determined by hydride generation atomic absorption spectrometry (HG-AAS)^25,49^.

One hundred Se-deficient mice were randomly divided into the following ten groups with 10 mice per group (in accordance to Table 3). The doses of Se were chosen according to the report by Peng^50^. Mice were fed a Se-deficient diet (<0.02 μg Se/g diet) during the experimental phase of the study. All administrations were conducted at nine o’clock for 7 consecutive days. All the doses were administered once daily (0.3 ml, i.g). Animals were sacrificed at the end of the experimental period. Blood samples were collected from the ophthalmic veins. The livers were quickly excised and rinsed with ice-cold normal saline. After excessive moisture was removed, liver tissues were weighed and packed in centrifuge tubes. The collected liver and blood samples were stored at −80 °C until use.

**Table 3:** Experimental mice groups related to bioactivity.

Groups	Contents (kg^−1^·BW^−1^) in normal saline (0.154 mol/L NaCl)
1	Normal saline
2	390 µg *R. palustris*
3	780 µg *R. palustris*
4	1560 µg *R. palustris*
5	390 µg SR (included 50 µg Se)
6	780 µg SR (included 100 µg Se)
7	1560 µg SR (included 200 µg Se)
8	80 µg SS (included 50 µg Se)
9	160 µg SS (included 100 µg Se)
10	320 µg SS (included 200 µg Se)

Se concentrations in whole blood and liver were determined by HG-AAS as described previously^49^. Liver tissue was homogenized by an automatic homogenizer. During the preparation, 0.5 g of each hepatic tissue was homogenized in nine-fold (w/v) ice-cold saline, and centrifuged at 15,000 g for 15 min. The supernatant was used for Se quantification and enzymatic assays. The activities of GSH-Px (Nanjing Jian Cheng, Nanjing, China) and TrxR (Beijing Solarbio, Beijing, China) in whole blood and liver were estimated using assay kits by spectrophotometry, according to the manufacturer’s instructions. Protein levels were determined by the Bradford assay with bovine serum albumin as a standard^48^.

### Protection against liver injury

Ninety mice were randomly divided into the following nine groups of 10 mice (in accordance to Table 4). The doses of Se were equivalent and according to that of Zhang^51^. All administrations were conducted at 9 o’clock in the morning for 14 consecutive days. All the doses were administered once daily (0.3 ml, i.g). On the fifteenth day, all the mice except the group1 and group2 received 0.3 ml of 1% CCl_4_/sunflower seed oil mixture (v/v) by intraperitoneal injection to induce acute hepatic injury, while the group1 and group2 received 0.3 ml of sunflower seed oil. After two hours, all the animals were fasted strictly, but drank water ad libitum as before. After CCl_4_ treatment (24 h), all animals were sacrificed to obtain blood and liver samples.

**Table 4:** Experimental mice groups show protection against liver injury.

Groups	Contents (kg^−1^·BW^−1^) in normal saline (0.154 mol/L NaCl)
1	Normal saline
2	1560 µg SR (included 200 µg Se)
3	Normal saline + CCl_4_
4	390 µg *R. palustris* + CCl_4_
5	780 µg *R. palustris* + CCl_4_
6	1560 µg *R. palustris* + CCl_4_
7	390 µg SR (included 50 µg Se) + CCl_4_
8	780 µg SR (included 100 µg Se) + CCl_4_
9	1560 µg SR (included 200 µg Se) + CCl_4_

Blood samples were collected from the ophthalmic veins of both eyes. The samples of blood were centrifuged at 1700 g for 30 min and supernatants were stored at 4 °C. The activities of AST, ALT, ALP, and LDH in serum were determined using commercial assay kits (Nanjing Jian Cheng, Nanjing, China), according to the manufacturer’s instructions. Livers were quickly excised, rinsed with ice-cold physiological saline, blotted with wet paper to remove excess moisture, and divided into two parts. The first part was homogenized in ice-cold physiological saline and centrifuged at 15,000 g at 4 °C for 15 min. The supernatant was used for biochemical assessments. The activities of GSH-Px, CAT, SOD and the levels of MDA in liver were measured using a commercial assay kit (Nanjing Jian Cheng, Nanjing, China). The second part of each liver [(1–2) mm × 5 mm × 10 mm] obtained from the left outer lobe of the organ was fixed in 10% buffered formaldehyde solution for histopathological examinations.

A conventional protocol^52^ was used for the histological examination of liver samples: livers were excised quickly and then fixed by direct immersion in a 0.1 M phosphate buffer (pH 7.4) with 4% paraformaldehyde for 24 h, dehydrated with a ethanol and toluene series, embedded in paraffin and sectioned as 4 μm-thick slices using a microtome (Leica RM2255, Solms, Germany). Five sections of each sample were stained with hematoxylin and eosin. Sections from the control and experimental samples were collected from similar tissue locations. Slides were examined with a light microscope (Olympus BX51, Tokyo, Japan).

### Statistical analysis

All experiments were performed in triplicate and the results were expressed as means ± SD. Data were analyzed using one-way ANOVA (SPSS 17.0). A p < 0.05 was considered as statistically significant.
